# Ovarian tissue cryopreservation after graft failure of allogeneic hematopoietic stem cell transplantation: first report and literature review

**DOI:** 10.3389/fendo.2024.1367241

**Published:** 2024-08-26

**Authors:** Jinghua Zhang, Xiaowei Li, Rong Liang, Shengnan Duan, Xin Yang, Yanru Hou, Li Tian

**Affiliations:** ^1^ Department of Obstetrics and Gynecology, Peking University People’s Hospital, Beijing, China; ^2^ Reproductive Medical Center, Peking University People’s Hospital, Beijing, China

**Keywords:** ovarian tissues cryopreservation, *in vitro* maturation, fertility preservation, hematopoietic stem cell transplantation, graft failure, aplastic anemia

## Abstract

**Background:**

Hematopoietic stem cell transplantation (HSCT) is an approach that has significantly improved the prognosis and survival of hematological patients. However, ovarian dysfunction and infertility following HSCT have gained increasing attention. Live births have been reported following ovarian tissue cryopreservation prior to HSCT and subsequent retransplantation of these tissues. Still, the feasibility of ovarian tissue cryopreservation (OTC) following graft failure (GF) of HSCT remains unknown. In this study, we report the first case of OTC following a GF of allogenic HSCT (allo-HSCT), as well as the cryopreservation of four MII oocytes via *in vitro* maturation with informed consent. Despite the lack of clinical outcomes after cryopreserved ovarian tissue retransplantation, we documented an interesting case in a woman after GF of allo-HSCT exhibiting functional ovaries and emphasized a clinical dilemma: whether OTC should be offered to women suffering from GF of HSCT.

**Case presentation:**

A 22-year-old woman with severe aplastic anemia who had suffered GF of allo-HSCT from her sibling brother [HLA allele match (7/10)] with a reduced dose conditioning regimen including fludarabine, cyclophosphamide, and antithymocyte globulin came to our reproductive center for fertility preservation, as she was about to receive the second allo-HSCT. We evaluated the ovarian reserve of this patient. Hormone assessments showed an anti-Müllerian hormone level of 3.921 ng/mL, a follicle-stimulating hormone level of 5.88 IU/L, a luteinizing hormone level of 10.79 IU/L, and an estradiol level of 33.34 pg/mL. Antral follicle counts accessed transvaginally showed 12–15 follicles. All assessments indicated a well-protected ovarian reserve. Due to the urgency of the second allo-HSCT, the patient decided to undergo ovarian cryopreservation. Laparoscopic surgery proceeded. Ovarian tissues were successfully cryopreserved using vitrification technology, and histologic evaluation demonstrated a follicle density of 20 per 2 × 2 mm^2^ biopsy with good viability. Four MII oocytes were obtained via *in vitro* maturation technology and cryopreserved. After the second HSCT, the patient relieved from aplastic anemia but suffered iatrogenic premature ovarian failure as predicted.

**Conclusion:**

OTC is applicable to fertility preservation in those undergoing GF of HSCT with benign hematological disorders and especially those who are about to receive the second HSCT.

## 1 Introduction

Aplastic anemia (AA) is a non-malignant hematologic disorder associated with bone marrow failure, characterized by pancytopenia (1). Treatments for AA typically consist of transfusion-related supportive care, antithymocyte globulin (ATG)-based immunosuppressive therapy, and hematopoietic stem cell transplantation (HSCT) (2). Among these, allogeneic hematopoietic stem cell transplantation (allo-HSCT) is playing an increasing role, as allo-HSCT from a matched sibling donor is recommended the first-line treatment for patients <40 years of age with severe aplastic anemia (SAA). Haploidentical HSCT is also recommended for relapsed/refractory AA patients who are unresponsive to immunosuppressive therapy (3, 4). In fact, allo-HSCT applied to AA treatment has achieved inspiring outcomes within the past decades as the long-term survival of patients is approaching 70%–80% (5). Graft failure (GF), a rare but severe complication of allo-HSCT, is defined as a lack of hematopoietic cell engraftment following HSCT, divided into primary GF (the absence of initial donor cells) and secondary GF (gradual loss of previously functioning graft) (6). Despite no global consensus on reversing this complication, the second HSCT (from either the same donor or another donor) is considered as a salvage strategy (6, 7).

However, ovarian function and fertility following allo-HSCT have attracted increasing attention. Between 65% and 84% of HSCT recipients suffered from ovarian failure (8), and only 0.6% were able to conceive successfully (9). Ovarian insufficiency following allo-HSCT is primarily associated with the intensity of conditioning (10). Graft-versus-host disease (GVHD) targeting ovaries are another contributing factor (11–13), which may account for donor T cell-mediated cytotoxicity in a cell–cell contact-dependent pattern (13). Our recent study has demonstrated the emerging role of iron overload and ferroptosis (a type of iron-dependent programmed cell death) in ovarian insufficiency (14), whereas chronic transfusion before HSCT and massive transfusion during HSCT typically cause iron overload in SAA patients, indicating the adverse effects of iron overload and ferroptosis on ovarian function of SAA patients post-HSCT. Overall, conditioning regimens, GVHD, and iron overload exert harm on ovaries and contribute to subfertility after allo-HSCT.

Currently, there are three primary techniques for female fertility preservation: embryo, oocyte, and ovarian tissue cryopreservation (OTC). Oocyte and embryo cryopreservation are recommended as established options for fertility preservation after puberty (15) but require controlled ovarian hyperstimulation. Therefore, these approaches are primarily applicable to subjects whose treatment can be postponed for approximately 2 weeks (16). OTC is an innovative approach that is capable of preserving fertility and restoring endocrine functions (15), acting as the only option for prepubertal children and women whose gonadotoxic treatments cannot be delayed (17). There have been reports of live births via OTC before HSCT and retransplantation after HSCT (18, 19). However, the feasibility of OTC after GF of allo-HSCT remains unclear.

In this report, we describe a practical dilemma when a 22-year-old SAA woman following GF of allo-HSCT who was about to undergo the second allo-HSCT came for fertility preservation consultation at our reproductive center. We thoroughly reviewed the associated literature and discussed about relevant issues. Finally, we cryopreserved ovarian tissues and four MII oocytes via *in vitro* maturation (IVM) for this patient with informed consent. Despite the lack of clinical outcomes after ovarian tissues retransplantation, we propose that young women with benign disorders suffering from GF of HSCT should not be excluded from OTC. We present relevant issues for consideration during this process.

## 2 Case report

This patient was a 22-year-old woman with SAA who suffered GF of allo-HSCT and was going to receive the second allo-HSCT. She was diagnosed with AA at age 6, presenting with dizziness and gingival bleeding. After a definite diagnosis, she was managed long-term on cyclosporin (CsA), androgens, and prednisone. During this period, her hemogram results showed a hemoglobin level between 6 g/dL and 8 g/dL and a platelet count of 10–20 × 10^9^/L; white blood cell and neutrophil counts were normal. When she was 18 years old, her condition deteriorated, as blood transfusions were required monthly to maintain her hemoglobin level between 6 g/dL and 7 g/dL, and allo-HSCT was recommended by hematologists. In September 2022, this patient underwent marrow and peripheral HSCT (marrow mononuclear cells 2.88 × 10^8^/kg, CD34 4.91 × 10^6^/kg) from her brother [HLA allele match (7/10), AB to AB]. The reduced dose conditioning (RIC) regimen encompassed fludarabine (FLU 30 mg/m^2^, −7 to −3), cyclophosphamide (Cy, 40 mg/kg, −5 to −2), and ATG (−4 to −1). GVHD prophylaxis consisted of CsA, mycophenolate mofetil (MMF), and short-course methotrexate (MTX). The patient suffered no GVHD according to clinical GVHD scores. The first HSCT was declared as secondary GF with gradual chimerism reduction. A second allo-HSCT from another donor was recommended by hematological specialists, and there was an unrelated donor with completely matched HLA. Given that a myeloablative conditioning regimen would be administered and premature ovarian insufficiency would be highly likely to occur, this young woman came for fertility-preserving consultation under the advice of hematologists.

**Figure 1:**
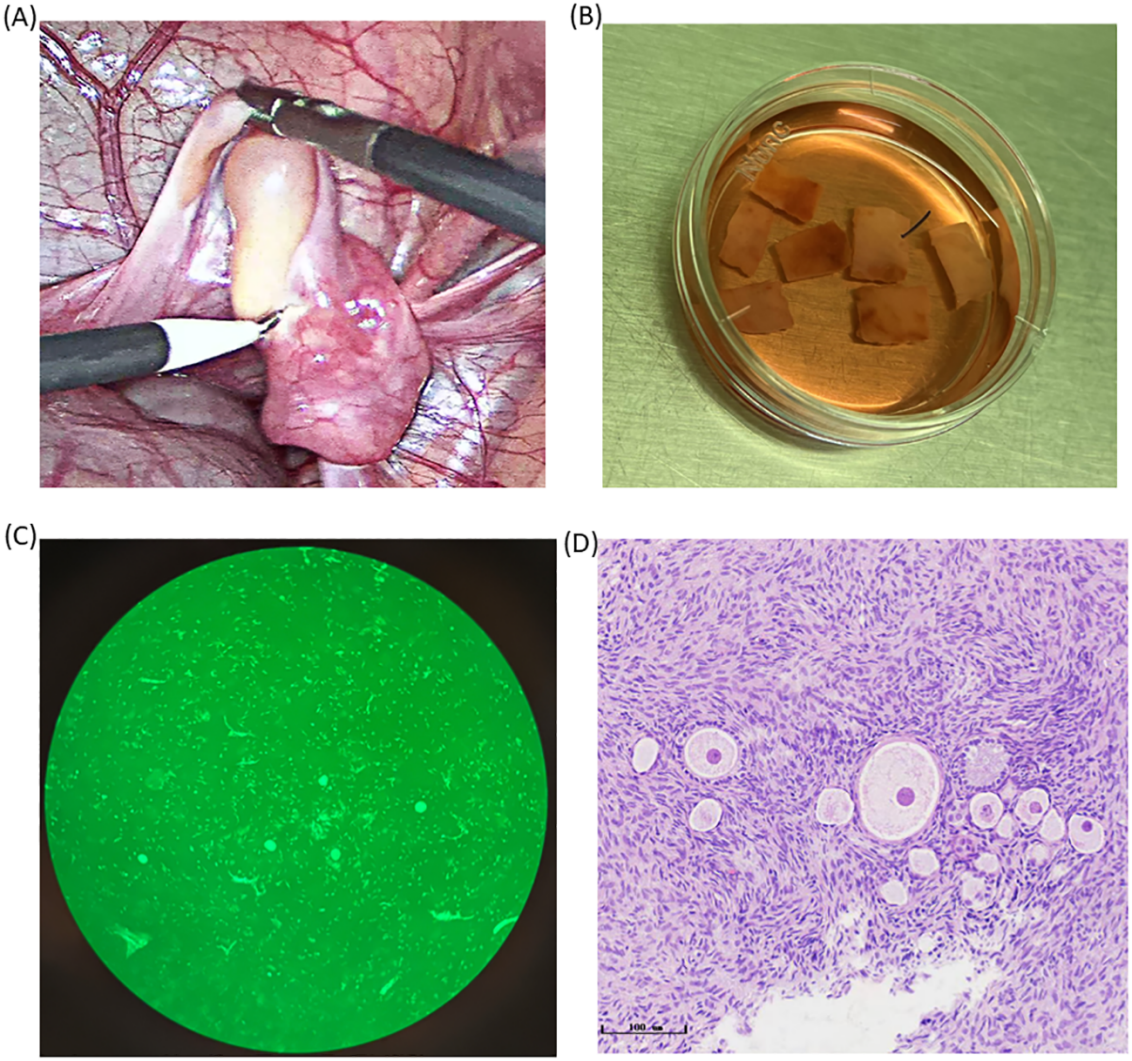
Photos of ovarian tissues during the OTC process. **(A)** The photo of the ovary during ovariectomy. **(B)** Ovarian tissue was transported to ovarian tissue Cryobank and was sliced for ovarian tissue preparation. **(C)** Primordial follicles scattered irregularly in the ovarian cortex as is shown by HE staining (bar = 50 μm). **(D)** Detection of follicular activity in the ovarian cortex.

We performed blood tests and transvaginal ultrasound on the patient. Hormone assessments showed an anti-Müllerian hormone (AMH) level of 3.921 ng/mL, a follicle-stimulating hormone (FSH) level of 5.88 IU/L, a luteinizing hormone (LH) level of 10.79 IU/L, a progesterone level <0.10 pg/mL, an estradiol level of 33.34 pg/mL, and a serum ferritin level of 11,505.0 ng/mL. Antral follicle counts assessed trans-vaginally indicated 12–15 follicles. Based on these results, the patient was in the follicular phase and exhibited a well-preserved ovarian reserve. In addition, according to this patient, she had a previous history of polycystic ovary syndrome without being treated. We offered the options of OTC and oocyte cryopreservation for this unmarried patient. She chose OTC due to the urgency of allo-HSCT and the demand for endocrine function recovery. On 01/04/2023, the patient received a platelet and blood transfusion, and the right ovary was removed by laparoscopy. Seven cortical slices (10 mm × 5 mm × 1 mm × 3 slices, 5 mm × 5 mm × 1 mm × 4 slices) were cryopreserved using vitrification technology, and Calcein-AM (MedChemExpress, the US) staining exhibited a follicle density of 20 per 2 × 2 mm^2^ biopsy with high viability (**Figure 1**). Additionally, we harvested four immature oocytes and vitrified four MII oocytes via IVM. The detailed process of vitrificated OTC can be found in **Supplementary Materials**. It was noted that the patient showed attenuated expression of hemagglutinin A and hemagglutinin B associated with her hematological disorder, making it challenging to identify her blood group. After confirming that her original blood group was AB Rh D positive, we prepared AB Rh D positive blood before surgery. The woman suffered postoperative fever with a maximal temperature of 38.8°C. Intravenous infusion of antibiotics was effective. The AMH level on April 6 was 2.746 ng/mL.

On 29/04/2023, the patient received the second allo-HSCT from an unrelated matched donor with a conditioning regimen of Bu, FLU, Cy, ATG, and GVHD prophylaxis of FK506, MMF, and MTX. This patient has been completely relieved of the primary hematopoietic disorder, suffered from amenorrhea, and adopted hormone replacement therapy until our most recent follow-up in July 2024. Hormone assessments were conducted 2 months, 4 months, and 1 year after the second allo-HSCT (**Table 1**), all of which indicated premature ovarian insufficiency.

**Table 1:** Timeline and major events of the patient.

Time	Event	AMH (ng/mL)	FSH ( IU/L)	LH (IU/L)	Estradiol (pg/mL)
12/09/2023	First allo-HSCT	–	–	–	–
28/03/2023	Fertility preservation consultation	3.921	5.88	10.79	33.34
01/04/2023	OTC	–	–	–	–
06/04/2023	Post-surgery	2.746	–	–	–
29/04/2023	Second allo-HSCT	–	–	–	–
03/07/2023	2 months after the second allo-HSCT	0.010	70.70	83.56	<20
22/09/2023	4 months after the second allo-HSCT	0.030	90.64	76.23	<20
09/03/2024	1 year after the second allo-HSCT	0.040	66.04	41.16	<20

AMH, anti-Müllerian hormone; FSH, follicle stimulating hormone; LH, luteinizing hormone; OTC, ovarian tissue cryopreservation; allo-HSCT, allogenic hematopoietic stem cell transplantation.

## 3 Discussion

In this report, we document an interesting case of a woman following GF of allo-HSCT who still exhibited well-functional ovaries and highlight a practical dilemma: whether to perform OTC for women who have undergone GF of allo-HSCT and will receive the second allo-HSCT. To our knowledge, this is the first case report of OTC for a patient following GF of allo-HSCT. We highlight the challenges faced and confirm the feasibility of OTC following GF of allo-HSCT and the necessity of OTC before the second allo-HSCT. Although fertility preservation is undoubtedly recommended before HSCT, patients are often not appropriately counseled to raise awareness of gonadal toxicity. This population suffering from gonadal toxic treatment should not be directly excluded from fertility preservation, especially those suffering from GF and are about to undergo the second HSCT, as accumulated gonadal toxicity makes fertility recovery almost impossible. There may be a growing number of women following HSCT demanding for fertility preservation with improved awareness of fertility preservation, suggesting that a novel issue worth examining yet has not been explored. We took the lead in proposing relevant issues that should be addressed, hoping to provide recommendations for daily practice.

One major issue is ovarian function, including destroying factors against ovaries and ovarian function in the present and future, which determine whether fertility preservation should be processed from the perspective from ovarian function. The factors impairing ovarian function on account of allo-HSCT are as follows.

The main factor accounting for ovarian dysfunction post-HSCT is conditioning, including high-dose myeloablative and RIC regimens. Myeloablative regimens are risky to ovarian failure, as >90% of these women suffer from gonadal dysfunction and a high rate of infertility (20). Alkylating agents like Cy, BU, and melphalan are the most toxic to ovaries (21), damaging actively dividing cells, including mature follicles and granulosa cells (21). When receiving BU and Cy in combination, the risk of complete premature ovarian failure is almost 100% (22). RIC was reported to mitigate the risk for ovarian insufficiency, with 68.1% of patients suffering from amenorrhea and 86.3% of cases suffering from impaired ovarian function (23). There have been reports of live births in patients undergoing RIC regimens (24). Therefore, conditioning regimens have adverse effects on ovaries, whereas the adverse effects of RIC are smaller than myeloablative regimens.

The second factor accounting for ovarian insufficiency post-HSCT is GVHD targeting ovaries. This phenomenon was first described in female mice injected splenocytes from allogeneic mice (12), and later in allo-HSCT mouse models (11, 13), in which ovarian disorders caused by GVHD following allo-HSCT were described as atrophic volumes (13), decreased AMH (11, 13), fewer numbers of retrieved oocytes upon superovulation (11), or induced ovulation (13), as well as lower numbers of naturally conceived newborns (11, 13) compared with syngeneic transplantation. Histopathological evidence demonstrated the infiltration of donor T cells in ovaries (11, 13), where donor CD8 T cells conferred cytotoxicity in a cell–cell contact-dependent pattern (13). GVHD prophylaxis with prednisone or clinically relevant GVHD prophylaxis with CSP or TAC restored AMH production and ovulation (13). It is noted that GVHD targeting ovaries was described in animal models, whereas attention to GVHD in humans primarily focused on the lungs, skin, gastrointestinal tract, liver, and genital tract (25, 26). This is likely because graft–versus–gonads are usually non-lethal and deficient in noticeable clinical manifestations. However, this does not mean gonads will not be attacked by transplants (11–13). Mouse models have directly demonstrated GVHD-associated ovarian injuries following allo-HSCT independent of conditioning (13). However, the adverse effects of GVHD on human ovaries after allo-HSCT have not been well studied, which might be overwhelmed by the devastating effects of conditioning (13). A previous study evaluating AMH levels in six young female long-term survivors after HSCT indicated that serum levels of AMH were restored in five patients with no or mild acute GVHD, but not in one patient with severe acute and chronic GVHD (27). This indicates the presence of human ovarian damage targeted by graft.

The third factor accounting for ovarian insufficiency and infertility post-HSCT is iron overload and ferroptosis. Iron overload is common among hematopoietic patients due to transfusion dependency of primary diseases or pre-successful implantation of hematopoietic stem cells during HSCT. In our latest study, we elaborated female infertility with spotlight on iron overload and ferroptosis. More specifically, iron overload triggered ferroptosis in oocytes, impaired oocyte meiosis and quality (28), and triggered apoptosis (28) and ferroptosis of granulosa cells (29), the specific molecular mechanisms of which include oxidative stress triggered by TFRC-mediated cellular iron increase, ferroptosis associated with mitophagy, Hippo-YAP-Nrf2 pathway activation, NF2-Hippo-YAP pathway inhibition, etc. (14). Additionally, the endocrine function of ovaries was attenuated by high levels of iron (28), manifested as lower estrogen and progestin, as well as irregular estrous cycles in iron-overloaded mouse models, the molecular mechanism of which might be associated with inhibition of HIF-1α and WNT signaling (14). These demonstrated ovarian insufficiency triggered by iron overload and ferroptosis.

When this patient came for fertility consultation, she exhibited well-preserved ovarian function. This might be attributed to lower gonadal toxicity of RIC, as well as minor GVHD associated with GVHD prophylaxis and GF. Individual heterogeneity is an important feature to consider also, including characteristics like well-preserved ovaries due to youthfulness and polycystic ovary syndrome. However, the patient was pending allo-HSCT with BU/FLU/Cy/ATG as the myeloablative conditioning regimen for the second time, accompanied by a large amount of transfusion and probable GVHD. Despite the lack of large-scale data on ovarian function after a second allo-HSCT, premature ovarian insufficiency would be predictive due to the cumulative toxicity of high-dose chemotherapy. Consequently, from the perspective of ovarian function, fertility preservation has been indicated.

The second issue is the prognosis of primary diseases, which determines whether the fertility preservation procedure should be launched from the perspective of the cost-effectiveness principle. GF is a life-threatening complication of allo-HSCT, and the second HSCT is recommended as a salvage option (30). Among SAA patients suffering from GF of allo-HSCT, the 4-year overall survival of the second umbilical cord blood stem cell transplantation was 38.5% (31) and the 5-year overall survival of the second SCT was 60.7% (32), 75% (31), and 95.7% (7), when the stem cells originated from bone marrow/peripheral blood, bone marrow, bone marrow, and peripheral blood of an HLA-matched sibling donor, respectively. This patient would apply peripheral blood stem cells from an HLA-matched unrelated donor to the second transplantation. Although no prognostic data have been reported, a favorable prognosis would be possible. Consequently, from the perspective of cost-effectiveness, fertility preservation could be indicated.

The third issue is the assessment of perioperative risks. Patients following GF tend to be weak due to unrecovered hematopoietic function and susceptibility to various infections. Moreover, allo-HSCT and primary hematopoietic diagnosis may trigger an abnormal expression of blood group antigens (33), making it difficult for blood type identification. This patient encountered an abnormal expression of blood group antigens and had a fever following surgery, highlighting the significance of management during the perioperative period.

In general, for women suffering from GF of allo-HSCT, if they have good ovarian reserve, favorable prognosis, and predictable ovarian failure and can tolerate perioperative risks, OTC should be considered. In fact, these may be coincident events in young women with benign hematological disorders receiving haploid transplantation. According to clinical research, non-malignancies (34), HLA disparity (6), RIC (30), and intensive T-cell depletion of the graft (35) are all risk factors of GF. RIC is usually applied to young patients without a related matched donor (4), and T-cell depletion of the graft commonly suggests minor GVHD, meaning that these patients are more likely to suffer from GF and good ovarian function following HSCT. We focus on benign diagnosis here, as malignant cases suffering from GF usually exhibit more unfavorable prognoses (34), and the ovaries may be contaminated by malignant cells (36). It is noted that the 1-year overall survival of AA patients after GF was 52.1% to 82.4% (37), sparing time for fertility preservation, and oocyte/embryo cryopreservation can be considered if there is time available for hyperstimulation.

Due to the urgency of gonadotoxic treatment and the demand of the patient, we cryopreserved ovarian tissues for this patient safely. This OTC was preliminarily evaluated as successful since cryopreserved ovarian tissues exhibited well-preserved ovarian reserves, indicated by a follicle density of 20 per 2 × 2 mm^2^ biopsy with good viability, four MII oocytes were also cryopreserved via IVM, and this patient had her AA relieved but suffered from iatrogenic ovarian failure after the second allo-HSCT.

In summary, OTC is a viable and safe approach for fertility preservation in young women with non-malignant hematological diagnoses who have suffered GF of HSCT. These patients are advised to consult reproductive specialists for fertility preservation prior to subsequent fertility impairment therapy, such as the second HSCT. Reproductive specialists should assess present ovarian function and gonadal toxicity of subsequent treatment to predict the prognosis and ovarian function. If the ovaries function well, favorable prognosis and iatrogenic ovarian failure are predicted, and then OTC may be applicable. Women after GF of HSCT should not be excluded from fertility preservation. We will go on to follow up with this patient, reporting the reproductive outcomes after retransplantation, with a view to providing references for analogous cases.

## 4 Conclusion

Young women with benign hematopoietic diagnoses who have suffered GF of HSCT should be taken into consideration of OTC. These patients are advised to consult reproductive specialists for fertility preservation before subsequent fertility impairment therapy, such as the second HSCT. If their ovaries function well, iatrogenic ovarian failure as well as a good prognosis of the primary disease is predicted, and high tolerance to the operation is evaluated, OTC with/without IVM is recommended as a viable and safe strategy for fertility preservation. We will follow up on the clinical outcomes pre- and post-cryopreserved ovarian tissue retransplantation, to provide references for analogous cases.
