# Supplementary material for: Ovarian tissue cryopreservation after graft failure of allogeneic hematopoietic stem cell transplantation: first report and literature review
Source: Front Endocrinol (Lausanne). 2024 Aug 26;15:1367241. doi: 10.3389/fendo.2024.1367241 (PMC11382424; doi:10.3389/fendo.2024.1367241)
Supplement: Supplementary file 1 [file DataSheet1.docx]

**Supplementary materials**

The excised ovary was transferred to the laboratory in cold (4 °C) medium. Before dissecting the ovarian cortical tissue for cryopreservation, we aspirated all visible follicles using an 18-gauge syringe attached to a 10-mL syringe. The aspirates together with COCs collected during ovarian tissue preparation, underwent biphasic IVM, which was performed in two steps using an IVM Media Kit (SAGE, USA). In the first step, COCs were cultured in groups of 10 in 500 µL of the IVM medium (IVM medium supplemented with 1 mIU/mL rFSH, 5 ng/mL insulin, 10 nM estradiol, 10 mg/mL HSA, and 25 nM CNP) under oil for 24 hours at 37 °C, under a 6% CO_2_ atmosphere. Following this 24-hour incubation, COCs were washed and transferred to the medium of 2nd step (SAGE IVM medium supplemented with 5 ng/mL insulin, 10 nM estradiol, 100 ng/mL human recombinant Amphiregulin, and 100 mIU/mL rFSH), and incubated for 30 hours under the same conditions. Then, ovarian tissues were cut from the equator of the ovary to obtain 1-mm-thick ovarian tissue slices. After removing the ovarian medulla, the ovarian cortex was cut into small pieces, and a total of 7 pieces of ovarian cortical slices (10 mm × 5 mm × 1 mm ×3 slices, 5 mm × 5 mm × 1 mm × 4 slices) were obtained. The cortical sections were put into cryopreservation solution 1, cryopreservation solution 2, and cryopreservation solution 3 (Cryotissue Vitrification Kit, KITAZATO, Japan) for 5, 5, and 15 minutes, respectively. After absorbing the excess cryopreservation solution with sterile gauze, ovarian slices were collected in sterile tubes and flash-frozen using liquid nitrogen.
